# Lead-free halide perovskite memristors for scalable crossbar arrays

**DOI:** 10.1186/s40580-025-00507-z

**Published:** 2025-08-25

**Authors:** Do Yeon Heo, Hyojung Kim

**Affiliations:** 1 Hydrogen Ion Materials Group, National Institute for Materials Science, Tsukuba, 305-0044 Japan; 2 Department of Semiconductor Systems Engineering, Sejong University, Seoul, 05006 Republic of Korea

**Keywords:** Halide perovskites, Lead-free, Memristor, Resistive switching memory devices, Crossbar array

## Abstract

Lead-free halide-perovskite memristors have advanced rapidly from initial proof-of-concept junctions to centimeter-scale selector-free crossbar arrays, maintaining full compatibility with CMOS backend processes. In these highly interconnected matrices, surface passivation, strain-relief interfaces, and non-toxic B-site substitutions successfully reduce sneak currents and stabilize resistance states. The Introduction section lays out the structural and functional basis, detailing phase behavior, bandgap tunability, and tolerance-factor-guided crystal design within Ruddlesden–Popper, Dion–Jacobson, vacancy-ordered, and double-perovskite frameworks, each of which is evaluated for its ability to confine filaments and reduce crosstalk in crossbar configurations. The following sections examine the characteristics of charge transport and the dynamics of ion migration, followed by a detailed outline of chemical and mechanical stabilization strategies in response to the high current densities and heat fluxes typical of large-area crossbars. The comparison of solution, vapor, and solid-state synthesis routes focuses on aspects such as film uniformity, grain-boundary control, and compatibility with flexible or heterogeneous substrates, all evaluated against the demanding uniformity requirements of multilevel crossbar programming. The principles of resistive switching and array architecture are elaborated upon, emphasizing the three-dimensional (3D) stacking of selector-integrated vertical nanowires and hybrid photonic-memristive layers as promising approaches to enhance bandwidth and reduce energy consumption per operation. By integrating sustainable chemistry with scalable crossbar engineering, these memories are set to provide ultra-dense, energy-efficient hardware that meets the performance demands of contemporary artificial intelligence accelerators while adhering to new regulations on hazardous materials in electronic devices.

**Graphical Abstract:**

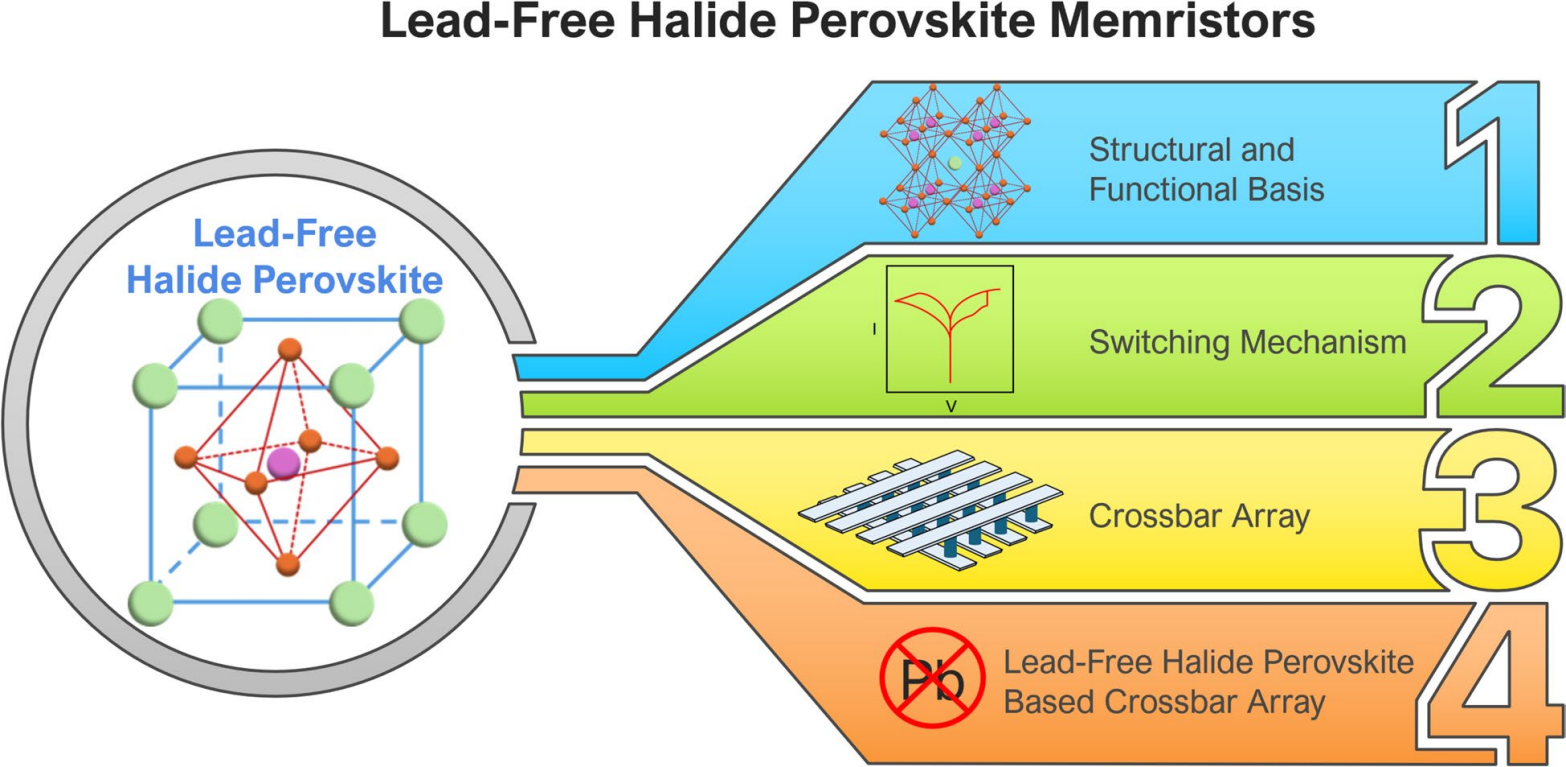

## Introduction

The rapid expansion of artificial intelligence inference, edge analytics, and the Internet of Things (IoT) has revealed distinct limitations in the data-transfer architecture of traditional computers [1–7]. In response to the growing requirements for speed and energy efficiency, halide perovskite materials are undergoing thorough research for a wide range of electronic applications, such as light-emitting diodes, gas and chemical sensors, low-temperature thin-film transistors, and resistive switching memories [8–14]. A crystalline framework that enables ultrafast electronic transport alongside field-driven ion migration presents appealing possibilities for integrating light emission, sensing, logic, and non-volatile storage into a single structure [15–19]. Halide perovskites processed through solution methods exhibit a soft ionic lattice, significant defect tolerance, and a band gap that can be tuned through composition. These characteristics allow for accurate regulation of threshold voltage, data-retention window, and mechanical flexibility while ensuring compatibility with large-area production on plastic substrates. Considering these benefits, it is noteworthy that most high-performance demonstrations continue to depend on lead-based ABX_3_ compounds [20–26]. The presence of lead raises significant toxicity issues, prompting regulating examination and accelerating moisture-related deterioration, which compromises long-term dependability. Thus, lead removal is one of the most important steps toward sustainable deployment. Ongoing studies concentrate on halide perovskite free from lead, utilizing alternatives such as tin (Sn), bismuth (Bi), and various mixed multivalent cations, often stabilized within two-dimensional (2D) Ruddlesden-Popper or Dion-Jacobson lattices [27–31]. Hydrophobic organic spacers are inserted into layered designs to prevent water intrusion, raise ion motion activation barriers, and inhibit phase segregation.

Memristors constructed using these substances demonstrate sub-volt switching capabilities, ON/OFF ratios exceeding 10^4^, and endurance exceeding ten thousand cycles, all accomplished via low-temperature solution processing. Additional difficulties arise when extending single-device success to wafer-scale crossbar arrays. Mechanical strain during bending, sneak-path currents, and cycle-to-cycle fluctuation can all reduce array-level dependability. These problems have started to be lessened by promising techniques such as grain-boundary passivation that smoothed local electric fields, threshold selectors in series, and built-in rectification from asymmetric band alignment [32–34]. This review initially outlines the crystal chemistry and low-temperature synthesis methods for lead-free perovskites, followed by an explanation of the microscopic switching mechanisms that determine essential device metrics. The following sections delve into the engineering practices of single memristors, covering aspects such as electrode selection and interface passivation. This is followed by discussions on array integration, which encompass selector design and the mitigation of sneak currents. The next focus is on evaluating reliability and environmental durability, followed by a conclusion that explores the potential for three-dimensional stacking, flexible substrates, and various data-driven applications.

## Halide perovskite

### Structural and chemical properties of halide perovskites

Halide perovskites are a class of materials defined by their ABX_3_ crystal structure, where A represents a monovalent cation such as cesium (Cs^+^), methylammonium (CH_3_NH_3_^+^, MA^+^), or formamidinium (NH_2_CH = NH_2_^+^, FA^+^), B is a divalent metal cation like lead (Pb^2+^) or tin (Sn^2+^), and X is a halide anion such as chloride (Cl^−^), bromide (Br^−^), or iodide (I^−^). This structural framework has enabled halide perovskites to achieve widespread recognition in optoelectronic applications, including solar cells, light-emitting diodes (LEDs), and photodetectors. Their remarkable properties, such as high absorption coefficients, tunable bandgaps, long carrier diffusion lengths, and efficient charge transport, have made them highly desirable for advanced technologies. More recently, halide perovskites have emerged as promising candidates for resistive switching memory devices due to their unique electronic characteristics.

**Fig. 1:**
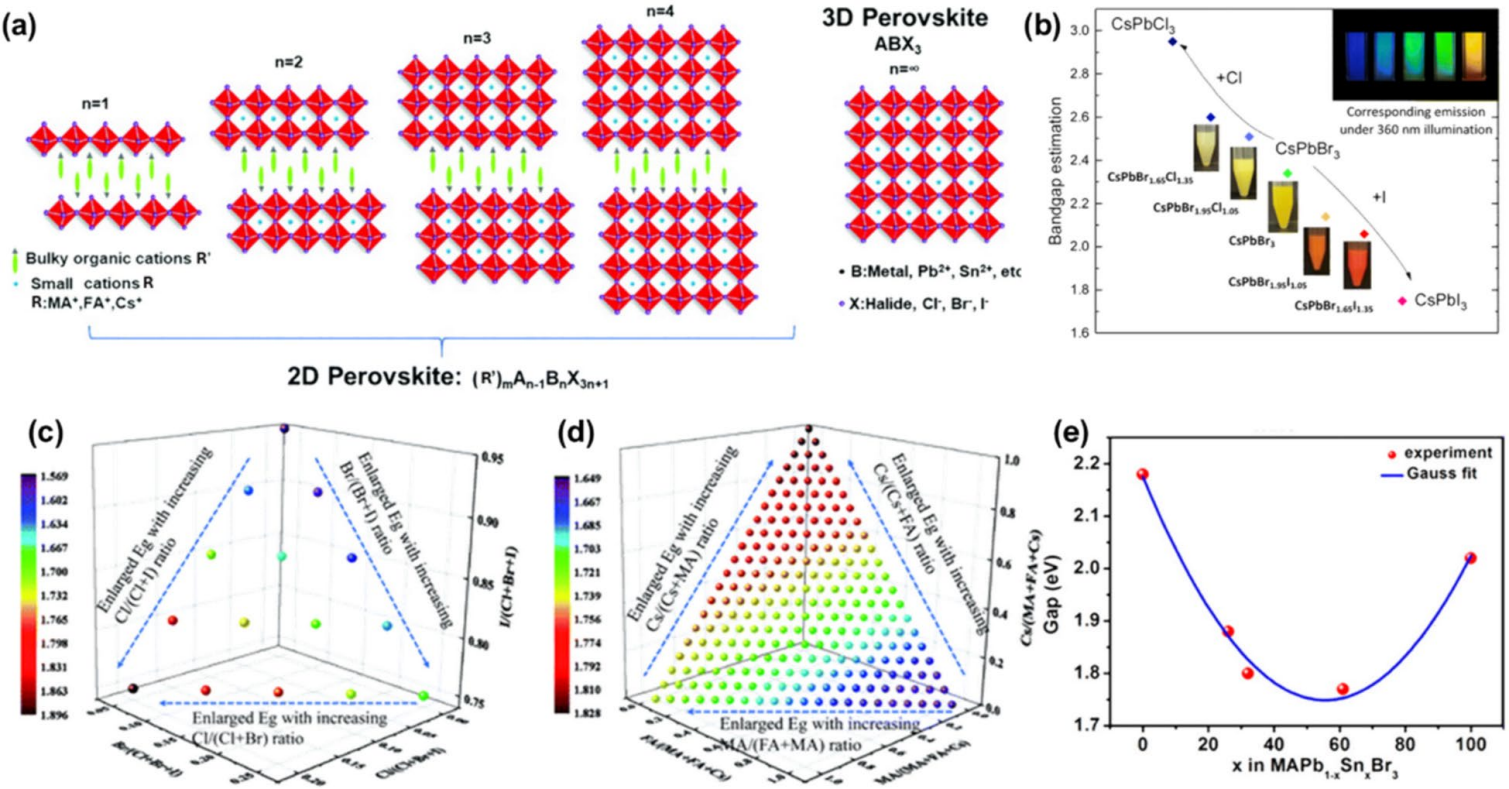
Structural characteristics and bandgap tunability of halide perovskites. **a** Architectural comparison between layered 2D perovskites (n = 1–4) and conventional 3D perovskites, illustrating the dimensional transition. Green arrows indicate bulky spacer cations (R’), while octahedral frameworks comprise metal cations (B: Pb^2+^, Sn^2+^) and halides (X: Cl⁻, Br⁻, I⁻). Reprinted with permission from [35]. Copyright 2020, The Royal Society of Chemistry. **b** Optical bandgap modulation in CsPb(Br/X)_3_ systems (X = Cl, I) determined through spectroscopic analysis. The inset displays photoluminescence of colloidal nanocrystals under UV excitation, demonstrating composition-dependent emission spanning blue to red. Reprinted with permission from [45]. Copyright 2023, Royal Society of Chemistry. **c** Three-dimensional visualization of computational bandgap predictions as a function of the halide composition (fixed cation ratio FA:MA:Cs = 0.75:0:0.25), with color gradient representing bandgap magnitude (eV). **d** Complementary bandgap prediction map showing the effect of cation stoichiometry variation at constant halide ratio (Cl:Br:I = 0.05:0.15:0.8). Reprinted with permission from [46]. Copyright 2021, The Royal Society of Chemistry. **e** Experimental band gap evolution in MAPb_(1-*x*)_Sn_*x*_Br_3_ with composition *x*, revealing a nonlinear relationship fitted to a Gaussian model. Reprinted with permission from [47]. Copyright 2018, American Chemical Society

One of the most intriguing aspects of halide perovskites is their ability to exist in various dimensionalities, including three-dimensional (3D), two-dimensional (2D), and quasi-2D structures. Each dimensionality offers distinct advantages and challenges, making the choice of structure critical for optimizing performance in specific applications. In the 3D halide perovskite structure, the BX_6_ octahedra are interconnected in all three spatial directions, forming a robust and continuous inorganic framework (Fig. 1a) [35]. The A-site cations occupy the cavities within this lattice, balancing the overall charge and stabilizing the structure. This fully connected network facilitates efficient charge transport throughout the crystal and results in relatively low exciton binding energies, which is advantageous for optoelectronic applications. However, the lack of organic spacers or hydrophobic barriers renders 3D perovskites highly susceptible to environmental factors such as moisture and oxygen. This vulnerability often leads to rapid degradation of both the crystal structure and device performance under ambient conditions.

In contrast, 2D halide perovskites are characterized by their layered architecture, in which the inorganic BX_6_ octahedral sheets are separated by bulky organic cations. These organic spacers, which are typically larger than the A-site cations in 3D perovskites, interrupt the continuous inorganic network and confine the perovskite structure to a single or few atomic layers. The general formula for 2D perovskites is often expressed as R_2_A_*n*−1_B_*n*_X_3*n*+1_ (Fig. 1a), where R is the organic spacer cation and n denotes the number of contiguous inorganic layers between spacers [35]. When n equals one, the structure consists of a single layer of BX_6_ octahedra, fully separated by organic cations. This configuration introduces strong quantum and dielectric confinement effects, resulting in a wider bandgap and higher exciton binding energy compared to 3D analogs. The presence of hydrophobic organic layers significantly enhances resistance to moisture and environmental degradation, making 2D perovskites much more stable under ambient conditions. However, the insertion of organic spacers also disrupts the continuity of the electronic structure, leading to reduced charge carrier mobility, particularly in the out-of-plane direction.

Quasi-2D halide perovskites occupy an intermediate position between the 3D and pure 2D forms. In these materials, the structure consists of multiple inorganic layers, typically two to five, stacked together and separated periodically by organic spacer cations. This results in a hybrid structure where the inorganic framework is partially preserved, allowing better charge transport than in pure 2D perovskites, while still benefiting from the enhanced stability provided by the organic spacers. The properties of quasi-2D perovskites can be finely tuned by controlling the thickness of the inorganic layers (i.e., the value of n in R_2_A_*n*−1_B_*n*_X_3*n*+1_), with larger *n* values approaching the characteristics of 3D perovskites [36]. As *n* increases, the bandgap narrows and the exciton binding energy decreases, leading to improved electronic performance [37]. At the same time, the periodic incorporation of organic spacers continues to offer protection against moisture and other environmental stressors, providing a balance between stability and charge transport.

The structural differences among 3D, 2D, and quasi-2D halide perovskites are not merely academic; they have profound implications for the materials’ optoelectronic properties and their suitability for device applications. In the context of resistive switching memory, these structural features determine key parameters such as environmental stability, ON/OFF resistance ratio, and device endurance. The continuous inorganic network in 3D perovskites is advantageous for charge transport but problematic for stability, while the layered architecture of 2D and quasi-2D perovskites offers improved resistance to degradation at the expense of some electronic performance. Quasi-2D perovskites have emerged as promising candidates for memory devices, as they combine the best aspects of both 3D and 2D structures, namely, enhanced stability and tunable electronic properties.

In summary, the structural versatility of halide perovskites, spanning from fully connected 3D networks to layered 2D and quasi-2D architectures, underpins their unique combination of properties. The structural differences between 3D, 2D, and quasi-2D halide perovskites are summarized in Table 1. We will discuss the characteristics of 3D, 2D, and quasi-2D halide perovskites in detail in Sect.  2.3. Understanding these structural distinctions is essential for the rational design of materials with optimized performance for resistive switching memory and other advanced electronic applications.

**Table 1:** Summary of structural and functional differences between 3D, 2D, and quasi-2D halide perovskites

Feature	3D perovskite	2D perovskite	Quasi-2D perovskite
Formula	ABX_3_	R_2_A_*n*−1_B_*n*_X_3*n*+1_	R_2_A_*n*−1_B_*n*_X_3*n*+1_
Dimensionality	Isotropic 3D network	Layered (Single inorganic sheet)	Hybrid (2–5 inorganic layers)
Bandgap	1.5–2.0 eV	> 2.0 eV	1.8–2.2 eV
Stability	Low (moisture/ heat sensitive)	High (hydrophobic spacers)	Moderate to high
Charge transport	Excellent (all directions)	Poor (out-of-plane)	Improved (in-plane delocalization)
Environmental Stability	Poor (moisture-sensitive)	High (hydrophobic layers)	Moderate to high
Ion migration control	Weak (fast ion mobility)	Strong (confined migration)	Controlled (n-dependent)

### Properties of halide perovskites

Halide perovskites have attracted extensive attention in materials science and device engineering due to their outstanding and tunable physical properties. Their unique combination of electronic, optical, and ionic characteristics originates from their flexible crystal structure and compositional diversity. In this section, we provide a comprehensive overview of the fundamental properties of halide perovskites, focusing on aspects that are particularly relevant to their application in resistive switching memory devices. We discuss their electronic and optical properties, ion migration phenomena, defect chemistry, environmental stability, and the impact of dimensionality and composition on these characteristics.

One of the most remarkable features of halide perovskites is their exceptional electronic transport properties. In high-quality single crystals and well-optimized thin films, halide perovskites can exhibit high charge carrier mobilities. This high mobility is attributed to the relatively defect-tolerant nature of the perovskite lattice, where the electronic states near the band edges are derived from the antibonding interaction between the B-site metal cation and the halide anion [38, 39]. As a result, shallow point defects such as vacancies and interstitials tend to form states that are close to the conduction or valence band edges, minimizing their impact on carrier transport [40]. This defect tolerance is a key reason why halide perovskites can achieve high performance even when fabricated by low-temperature and solution-based processes, which typically introduce more disorder compared to conventional semiconductors [40, 41].

In addition to high carrier mobility, halide perovskites are known for their long carrier diffusion lengths, which can reach several micrometers in optimized films [42, 43]. The long diffusion length is a consequence of both high mobility and long carrier lifetimes, which are made possible by the low density of deep trap states in the material [44]. This property is particularly advantageous for optoelectronic applications, such as solar cells and photodetectors, where efficient charge extraction is essential for high device performance. In the context of resistive switching memory, long carrier diffusion lengths can influence the spatial extent of conductive filaments or other switching regions, potentially affecting device endurance and uniformity.

The optical properties of halide perovskites are equally impressive. These materials typically exhibit strong and broad absorption in the visible region. The bandgap of halide perovskites is highly tunable through compositional engineering, allowing for precise control over the absorption edge and emission wavelength. For example, by varying the halide composition to chloride, bromide, and iodide, the bandgap can be tuned from the ultraviolet to the near-infrared. Figure 1b presents derived optical bandgap energies of CsPbBr_3−*y*_X_*y*_ (X = Cl, I) [45]. The bandgap of CsPbBr_3−*y*_X_*y*_ is tunable from 2.95 eV (CsPbCl_3_) to 2.06 eV (CsPbBr_1.65_I_1.35_) via anion exchange, demonstrating precise compositional control over optoelectronic properties. This trend was also revealed through machine learning algorithms. As shown in Fig. 1c, the bandgap of FA_0.75_Cs_0.25_Pb(Cl_1−*x*−*y*_Br_*x*_I_*y*_)_3_ perovskites is highly tunable through halide composition [46]: increasing Cl content (0–15%) elevates the bandgap from 1.59 eV to 1.83 eV, while raising Br (5–20%) enhances it from 1.63 eV to 1.72 eV. Similarly, substituting different A-site or B-site cations can further modify the band structure. Figure 1d illustrates the bandgap modulation in pure iodide perovskites (Cs_*α*_FA_*β*_MA₁_−*α*−*β*_PbI_0.8_Br_0.15_Cl_0.05_), where increasing Cs content widens the bandgap, while higher FA ratios reduce it [46]. For chloride-based systems, Cs and FA exert comparable influence on bandgap tuning, highlighting the critical role of A-site cation engineering in tailoring the optoelectronic properties. Figure 1e shows the tunable bandgap of the MAPb_*x*_Sn_1−*x*_Br_3_ with increasing Sn^2+^ [47]. In 2D and quasi-2D perovskites, quantum and dielectric confinement effects lead to even larger bandgaps and higher exciton binding energies compared to their 3D counterparts [48]. This tunability is crucial for tailoring the electronic and switching properties of perovskite-based memory devices.

Another important aspect of halide perovskites is their ionic conductivity and the associated phenomenon of ion migration. Unlike conventional semiconductors, halide perovskites are mixed ionic-electronic conductors, meaning that both electrons (or holes) and ions can move through the lattice under an applied electric field. The most mobile ionic species are typically halide anions (Cl^−^, Br^−^, I^−^), although migration of A-site and B-site cations has also been observed under certain conditions [49, 50]. Ion migration can lead to a variety of effects in devices, including hysteresis, current–voltage instability, and long-term degradation [51]. However, in resistive switching memory devices, controlled ion migration can be harnessed to form and dissolve conductive filaments or to modulate the interfacial properties, enabling non-volatile resistive switching behavior [52]. Understanding and controlling ion migration is therefore central to optimizing the performance and reliability of perovskite-based memory devices.

The defect chemistry of halide perovskites is another critical factor influencing their properties. As mentioned earlier, the perovskite lattice is relatively tolerant to point defects, but the concentration and type of defects can still have significant effects on device performance. For example, halide vacancies can act as shallow donors or acceptors, modifying the carrier concentration and Fermi level position [53, 54]. In some cases, defect clustering or phase segregation can occur, particularly under prolonged electrical bias or illumination, leading to performance degradation [55, 56]. Strategies to control defect formation include careful selection of precursor stoichiometry, incorporation of passivating agents, and post-synthetic treatments such as annealing or surface modification [57].

Environmental stability is a well-known challenge for halide perovskites, especially those based on lead and iodide. Exposure to moisture, oxygen, heat, or light can trigger chemical decomposition, phase transitions, or ion migration, all of which degrade device performance over time [58, 59]. The instability is most pronounced in 3D perovskites, where the absence of hydrophobic organic layers leaves the inorganic lattice vulnerable to attack. In contrast, 2D and quasi-2D perovskites incorporate bulky organic spacer cations that act as barriers against moisture and other environmental stressors [60]. Experiments show that 3D CsPbI_3_ memory devices exhibit rapid performance degradation in just five days, while quasi-2D (PEA)_2_Cs_3_Pb_4_I_13_ devices maintain high ON/OFF ratios for two weeks [61]. This enhanced stability is a major reason why 2D and quasi-2D perovskites are being actively explored for applications requiring long-term operational reliability, such as memory devices.

The dimensionality of the perovskite structure plays a central role in determining its properties. In 3D perovskites, the continuous network of corner-sharing octahedra facilitates isotropic charge transport and relatively low exciton binding energy. However, the lack of organic spacers makes these materials more susceptible to environmental degradation [62]. In 2D perovskites, the introduction of organic spacers between inorganic layers leads to quantum and dielectric confinement, resulting in larger bandgaps, higher exciton binding energies, and reduced out-of-plane charge mobility [37]. Quasi-2D perovskites, which contain multiple inorganic layers separated by organic spacers, offer a compromise between the high stability of 2D structures and the superior charge transport of 3D structures [63]. By tuning the thickness of the inorganic layers (i.e., the value of n in the general formula R_2_A_*n*−1_B_*n*_X_3*n*+1_), it is possible to engineer materials with tailored optoelectronic and stability characteristics. Ribeiro et al*.* investigated the structural, energetic, and optoelectronic properties of 2D FPEA_2_(MA)Pb_*n*−1_I_3*n*+1_ (FPEA = 4-fluorophenylethylammonium) perovskite thin films as a function of the inorganic layer thickness (*n* = 1–4) using ab initio density functional theory (DFT) calculations [64]. Their results demonstrate that increasing *n* reduces the optical bandgap and enhances light absorption, approaching the optoelectronic performance of 3D perovskites. Simultaneously, higher *n* values improve structural and thermodynamic stability, achieving an optimal balance between device efficiency and environmental robustness.

Compositional engineering provides another powerful tool for tuning the properties of halide perovskites. Substituting different A-site cations, such as replacing methylammonium with formamidinium or cesium, can enhance thermal stability and modify the bandgap [65]. B-site substitution, such as replacing lead with Sn, Bi, or copper, enables the development of lead-free perovskites that are more environmentally benign. Ali et al*.* proposed 20 new perovskites by introducing Ge, Sn, Ca, and Sr as Pb-free B-sites [66]. The Pb-free perovskites exhibited direct band gaps of 1.42–1.77 eV and high absorption coefficients (10^5^ cm^−1^). The choice of halide anion also profoundly affects the optical and electronic properties, with chloride, bromide, and iodide offering a wide range of bandgaps and lattice parameters [65, 67]. Mixed-halide and mixed-cation perovskites have been developed to further optimize device performance and stability, often exhibiting superior properties compared to their single-component counterparts.

The interplay between structure, composition, and dimensionality in halide perovskites gives rise to a rich landscape of physical properties that can be precisely tailored for specific applications. For resistive switching memory devices, key parameters such as ON/OFF resistance ratio, switching speed, endurance, and retention are all influenced by the underlying material properties. For example, a larger bandgap, as found in 2D perovskites, can suppress leakage currents and enhance the ON/OFF ratio, while high ionic conductivity can facilitate the formation and dissolution of conductive filaments during switching. Loizos et al*.* experimentally demonstrated that a 2D (perfluorobenzyl)ammonium perovskite capping layer on top of a 3D mixed halide perovskite contributes to reducing the leakage current and improving the ON/OFF ratio (10^3^ → 10^4^) [68]. Achieving the optimal balance between electronic, ionic, and environmental properties is therefore essential for the successful implementation of halide perovskites in memory technologies.

Recent advances in characterization techniques have provided deeper insights into the fundamental properties of halide perovskites. Techniques such as time-resolved photoluminescence (TRPL) and impedance spectroscopy (IS) quantify carrier lifetimes and ion migration activation energies, which directly correlate with switching endurance and ON/OFF resistance ratios [69]. Scanning probe microscopy (SPM) tools, including Kelvin Probe Force Microscopy (KPFM), map nanoscale ion migration pathways and interfacial potential shifts under operational biases, elucidating filament formation dynamics in Ag/perovskite/Pt devices [70]. Photoemission electron microscopy (PEEM) further identifies defect-mediated trapping zones at grain boundaries [71]. Pump-push-photocurrent spectroscopy resolves metastable trap states (*τ*_*trap*_ ≈ 1–10 μs) that contribute to resistance drift [72], while transient ion drift (TID) measurements distinguish between vacancy-mediated and interstitial halide migration mechanisms [73]. These techniques collectively highlight the intricate interplay between electronic and ionic processes, underscoring the need for in situ multimodal approaches to optimize material design.

An elevated halide-vacancy migration barrier inhibits unregulated filament growth, consequently enhancing the overall endurance of resistive switching cycles. Tuning the band gap to decrease the metal/semiconductor injection barrier effectively lowers the threshold voltage. Conversely, intentionally increasing the gap can elevate the threshold, especially when prioritizing long-term data retention. Furthermore, the presence of passivated grain boundaries and reduced defect densities contributes to the stabilization of local electric fields, thus narrowing the distribution of threshold voltages throughout extensive arrays.

In summary, halide perovskites exhibit a unique combination of high carrier mobility, long diffusion lengths, strong optical absorption, tunable bandgaps, and mixed ionic-electronic conductivity. Their defect-tolerant nature and compositional flexibility enable the fabrication of high-performance devices using scalable, low-cost methods. However, challenges related to environmental stability, ion migration, and defect control must be addressed to fully realize their potential in resistive switching memory applications. Ongoing research into the fundamental properties of halide perovskites, combined with advances in material synthesis and device engineering, is expected to drive further progress in this exciting field.

### 2D halide perovskites

The practical application of halide perovskites in resistive switching memory and other advanced devices depends fundamentally on the interplay between their structural dimensionality, phase chemistry, and environmental robustness. While 3D halide perovskites have been at the forefront of research for their outstanding optoelectronic performance, their inherent instability under operational and environmental stress has become a critical bottleneck for real-world deployment. In contrast, 2D perovskites, especially those with Ruddlesden-Popper (RP) and Dion-Jacobson (DJ) phase architectures, have emerged as promising alternatives, offering a unique balance between electronic functionality and long-term resilience. This section provides a focused, in-depth comparison of these material classes, highlighting why the transition from 3 to 2D structures is not merely a matter of academic curiosity but a necessity for next-generation memory technologies.

#### Structural motifs and environmental stability of 2D halide perovskites

3D halide perovskites, typified by the general formula ABX_3_, are characterized by a continuous network of corner-sharing BX_6_ octahedra. This three-dimensional connectivity is the origin of their high carrier mobility, long carrier diffusion lengths, and efficient charge extraction, which have enabled rapid advances in solar cells, LEDs, and photodetectors. However, this same structural openness exposes the inorganic lattice to environmental agents such as water, oxygen, and heat. In practical terms, this means that 3D perovskites are acutely sensitive to moisture. MAPbI_3_ undergoes hydrolysis under humid conditions (85% RH), forming hydrated intermediates such as PbI_2_·H_2_O within 200 min, which irreversibly decompose into PbI_2_ after prolonged exposure (400 min) [74]. Similarly, CsPbBr_3_ reacts with water to yield PbBr_2_ and CsBr, as confirmed by in situ X-ray diffraction and mass loss analysis [75]. This process is not reversible under typical device conditions, and it leads to a rapid loss of optoelectronic function. The problem is further compounded by the volatility of organic A-site cations such as methylammonium, which can evaporate or decompose under illumination or thermal stress, leaving behind a defective and non-conductive lattice [74, 76].

Thermal instability is another major challenge for 3D perovskites. At elevated temperatures, typically above 85℃, halide segregation becomes pronounced, especially in mixed-halide compositions. This phase separation is often accompanied by the migration of lead ions and the release of corrosive byproducts such as hydrogen iodide or hydrogen bromide gases. These processes not only degrade the active layer but can also corrode electrodes and encapsulation materials, leading to catastrophic device failure. In the context of resistive switching memory, the high mobility of halide vacancies (such as I^−^ or Br^−^) and metal cations (notably Pb^2+^) under an applied electric field results in uncontrolled filament growth, hysteresis, and eventual rupture. As a result, 3D systems show poor reversibility of halide segregation in the dark, leading to irreversible performance loss[77]. Recognizing these limitations, researchers have turned to 2D perovskites, which introduce organic spacer cations into the lattice to create layered architectures. Two main structural motifs dominate the field: Ruddlesden-Popper (RP) and Dion-Jacobson (DJ) phases. Both are based on the principle of separating inorganic perovskite slabs with organic layers, but they differ fundamentally in the nature of their organic spacers and the resulting interlayer interactions.

**Fig. 2:**
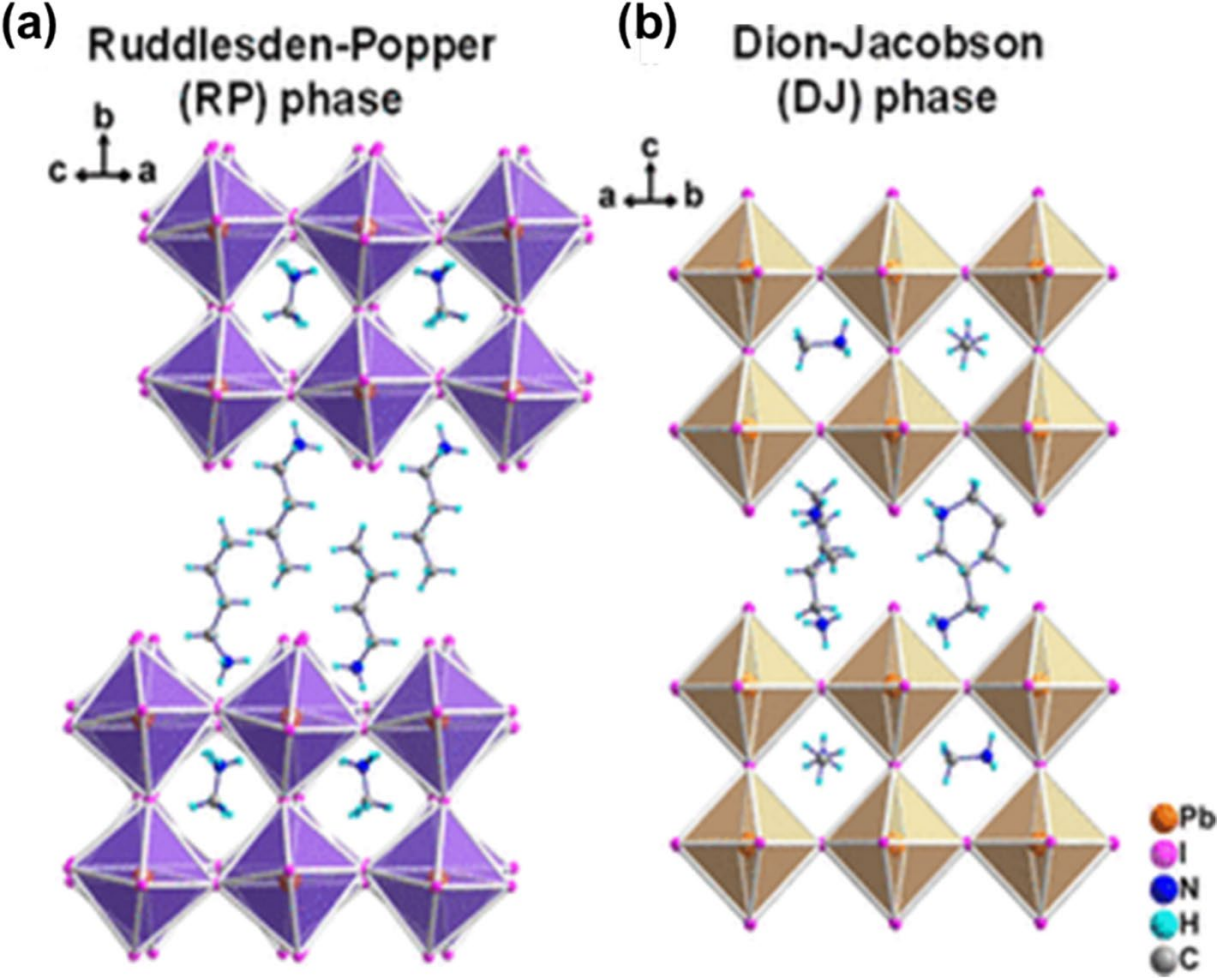
Crystal structures of two principal 2D halide perovskite phases along the ⟨100⟩ orientation. **a** Ruddlesden-Popper (RP) phase characterized by staggered inorganic octahedral layers with monovalent organic spacer cations forming bilayers between them. **b** Dion-Jacobson (DJ) phase featuring eclipsed (directly aligned) inorganic octahedral layers interconnected by divalent diammonium organic spacers that form covalent-like bonds with adjacent layers. Reprinted with permission from [78]. Copyright 2021, American Chemical Society

The RP phase is defined by the general formula R'_2_A_*n*−1_B_*n*_X_3*n*+1_, where R' is a bulky monoammonium cation, such as phenethylammonium (PEA^+^) or butylammonium (BA^+^), and n is the number of contiguous inorganic layers [78]. In this structure, the organic spacers are held between perovskite slabs primarily by van der Waals forces shown in Fig. 2a. This arrangement introduces hydrophobic barriers that slow the ingress of water molecules and thus dramatically improve moisture stability compared to 3D analogs. In Yang’s study, (PEA)_2_PbI_4_, a prototypical *n* = 1 RP perovskite, retains over 86% of its initial photoluminescence intensity after 96 h of storage at 65 ± 5% relative humidity, whereas MAPbI_3_ under the same conditions degrades completely within 96 h [79]. The hydrophobic organic spacer cations in 2D perovskites act as physical barriers, significantly slowing water ingress compared to 3D perovskites. This barrier effect stems from the non-polar nature of organic layers (e.g., alkyl chains or fluorinated groups), which repel polar water molecules and confine moisture interactions to the perovskite surface [80]. However, the weak van der Waals bonding between organic and inorganic layers in RP phases also introduces mechanical fragility. Under prolonged thermal or electrical stress, these layers can delaminate or slide past each other, leading to phase separation and a gradual decline in device performance [81].

The DJ phase, on the other hand, employs diammonium cations such as propane-1,3-diammonium (PDA^2+^) or hexane-1,6-diammonium (HDA^2+^) as spacers, resulting in the general formula R''A_*n*−1_B_*n*_X_3*n*+1_ [78]. Unlike the RP phase, the DJ structure features covalent-like bonding between the diammonium spacers and the inorganic slabs shown in Fig. 2b, eliminating the van der Waals gap and reducing the interlayer distance to less than 5 Å [82]. This compact, strongly bonded arrangement not only enhances mechanical robustness but also further suppresses the formation and migration of halide vacancies. In practical terms, DJ perovskites such as (BDP)CsPbBr_3_ have demonstrated no detectable phase segregation for 28 days under the air with 45% humidity at room temperature compared to cubic CsPbBr_3_ [83]. It is attributed to both the reduced free volume for water ingress and the higher activation energy required for ion migration across the covalently bonded layers [84].

#### Resistive switching mechanisms and memory performance in 2D and quasi-2D perovskites

The implications of these structural differences are profound when it comes to device performance, especially in resistive switching memory. In 3D perovskites, the high mobility of ionic species leads to filamentary switching mechanisms that are inherently unstable. The formation and rupture of conductive filaments are stochastic processes, resulting in large resistance fluctuations and poor endurance. In contrast, the layered structure of 2D perovskites, and especially the strong interlayer bonding in DJ phases, confines ion migration to in-plane directions and suppresses the formation of extended filaments. This results in more uniform switching events, higher ON/OFF resistance ratios, and vastly improved cycling stability. According to Loizos’ study, the F-BNA-based RP system displayed a prolonged cycling endurance of 10^4^ cycles and retention time of 5 × 10^3^ s, whereas the F-PDMA-based DJ perovskite devices achieved 5 × 10^3^ cycles and 3 × 10^3^ s retention, significantly surpassing the control device’s 1.2 × 10^3^ cycles and 1.2 × 10^3^ s retention [68]. This contrast underscores the superior stability of layered perovskites, with DJ phases benefiting from suppressed ion migration via covalent-like interlayer bonds.

Another critical advantage of 2D perovskites is their ability to accommodate quasi-2D structures, in which multiple inorganic layers (*n* = 2–5) are separated by organic spacers. These quasi-2D phases bridge the gap between the superior charge transport of 3D perovskites and the environmental stability of pure 2D structures. For example, (*i*BA)_2_(MA)_*n*−1_Pb_*n*_I_3*n*+1_ (*n* = 4) exhibits a responsivity of 117.09 mA/W while retaining excellent humidity stability for 60 days and ON/OFF ratios exceeding 4.0 × 10^2^ [85]. The reduced quantum confinement in quasi-2D structures lowers exciton binding energies and enhances free carrier generation, making them particularly attractive for high-performance memory and optoelectronic devices [86].

The environmental stability of 2D perovskites extends beyond moisture resistance. The strong interlayer bonding in DJ phases confers exceptional thermal and operational robustness. For example, DJ perovskite solar cells with slight-interlayer-displacement architectures retain over 90% efficiency after 4000 h at 85 ℃ and 90% relative humidity, conditions under which 3D perovskites rapidly degrade [87]. This stability stems from suppressed halide vacancy formation and a higher activation energy for ion migration in DJ structures. Furthermore, the compact, aligned layers of DJ phases minimize phase segregation and grain boundary defects, common failure modes in 3D perovskites. It is also important to note that the improved stability and performance of 2D perovskites are not achieved at the expense of versatility. The chemistry of the organic spacer cations, the thickness of the inorganic layers (*n* value), and the choice of metal and halide ions can all be independently tuned to optimize device characteristics for specific applications [88]. For example, by varying the length and functionality of the organic spacer, researchers can control not only the hydrophobicity and mechanical properties of the film but also the electronic coupling between layers and the overall band structure. This tunability is especially pronounced in quasi-2D perovskites, where the distribution of n phases can be engineered to achieve a desired balance between charge transport and stability [89, 90]. Advanced processing techniques, such as solvent engineering and thermal annealing, enable uniform *n*-phase distributions in quasi-2D perovskites, reducing defects and improving performance.

The role of defects and grain boundaries in 2D perovskites differs fundamentally from 3D analogs. In 3D perovskites, grain boundaries act as pathways for moisture ingress and ion migration, accelerating degradation. For example, polycrystalline MAPbI_3_ exhibits severe ion migration along grain boundaries, causing hysteresis and device failure under operational stress [91]. In 2D perovskites, the layered architecture restricts defect propagation. The organic spacer cations physically isolate inorganic layers, localizing defects (e.g., halide vacancies) and blocking moisture penetration [92, 93]. DJ phases further enhance stability through covalent-like interlayer bonds. While specific studies on DJ trap states are limited, the suppressed ion migration and minimized grain boundaries in DJ structures inherently reduce non-radiative recombination [94].

The advantages of 2D perovskites are further highlighted in the context of resistive switching memory. In these devices, the ability to control and confine ion migration is essential for achieving reliable, non-volatile switching behavior. The weakly bonded layers in RP phases already provide a significant improvement over 3D perovskites by restricting ion movement to the plane of the inorganic slabs. However, the DJ structure takes this a step further by essentially blocking out-of-plane ion migration altogether. As a result, DJ-based memory devices exhibit highly uniform switching thresholds, minimal cycle-to-cycle variation, and exceptionally long operational lifetimes. Recent studies have demonstrated that DJ perovskite capping layers in mixed-dimensional heterostructures suppress out-of-plane ion migration, enabling highly uniform resistive switching. For example, DJ-phase devices exhibit more than 10^3^ cycles with minimal resistance drift under continuous bias, outperforming 3D analogs in both endurance and environmental stability [68].

Another key area where 2D perovskites excel is in the development of lead-free, environmentally benign memory technologies. The ability to substitute lead with Sn, Bi, or other less toxic metals is greatly enhanced in the 2D framework, particularly in DJ phases [95]. The compact, strongly bonded structure of DJ perovskites stabilizes the oxidation state of alternative B-site cations, reducing their tendency to undergo unwanted redox reactions [89]. This is a crucial advantage for real-world applications, as it addresses both regulatory concerns and the long-term reliability of the devices. For instance, Bi-based DJ perovskites have demonstrated not only high endurance and data retention but also resistance to environmental degradation, making them strong candidates for sustainable electronics [96]. More details are covered in Sect. 2.4.

The integration of 2D perovskites into practical memory architectures also benefits from their compatibility with flexible substrates and low-temperature processing [97, 98]. Unlike many traditional oxide-based memory materials, which require high-temperature annealing and are thus incompatible with plastics and other flexible supports, 2D perovskites can be processed from solution at temperatures below 100℃ [98]. This opens the door to a wide range of applications in wearable electronics, flexible displays, and other emerging technologies where mechanical flexibility and low-cost manufacturing are essential.

#### Challenges and future prospects

Despite these advances, several challenges remain before 2D perovskite-based memory devices can achieve widespread commercialization. One of the primary hurdles is the scalability and reproducibility of high-quality 2D films, particularly for DJ phases [97]. While laboratory-scale demonstrations have shown promising results, translating lab-scale success to industrial-scale manufacturing requires innovation in precursor chemistry, deposition techniques, and post-processing protocols. Additionally, the long-term operational stability of lead-free 2D perovskites, especially those based on Sn or Bi, must be rigorously evaluated under accelerated aging conditions to ensure they meet the stringent requirements of industrial memory applications.

Another area of ongoing research is the optimization of the interface between the perovskite layer and the device electrodes. Studies demonstrate that grain boundary engineering in DJ perovskites reduces halide ion migration pathways, improving resistive switching uniformity. The choice of electrode material and interface design critically impacts switching behavior and endurance. For DJ perovskites, robust electrode contacts are essential to avoid side reactions. Metastable DJ phases with asymmetric organic spacers reduce energy barriers for charge transport, enabling low-resistance interfaces [99]. Recent advances include self-assembled monolayers (SAMs) and fluorinated buffer layers, which suppress moisture ingress and filament overgrowth. For instance, perfluoro-phenylene dimethylammonium (F-PDMA) capping layers improve endurance by a factor of four compared to 3D analogs [68].

Looking forward, the unique combination of structural tunability, environmental robustness, and electronic versatility offered by 2D perovskites, especially DJ phases, positions them at the forefront of next-generation resistive switching memory technology. Their ability to overcome the critical limitations of 3D perovskites, including moisture sensitivity, thermal instability, and uncontrolled ion migration, marks a paradigm shift in the design of durable, high-performance, and potentially lead-free memory devices. As research continues to refine the synthesis, processing, and integration of these materials, it is likely that 2D perovskites will play a central role in the evolution of sustainable and scalable electronic memory.

Despite the clear advantages of quasi-2D perovskites in balancing stability and charge transport, one critical challenge remains: phase heterogeneity arising from inconsistent distribution of the layer number *n*. During solution processing, a broad range of *n*-phase domains can form spontaneously due to kinetics-limited crystallization, leading to spatially varying bandgaps, trap densities, and ion migration behaviors across the film [100, 101]. As a result, memristor devices based on quasi-2D systems may exhibit limited switching uniformity, endurance degradation, and variability in resistive states. To address these issues, recent studies have focused on phase control via solvent engineering and annealing protocols. For instance, the use of high-boiling-point solvents combined with antisolvent dripping has been shown to narrow the *n*-phase distribution by encouraging thermodynamically stable growth. Moreover, controlled annealing—such as stepwise temperature ramping or gradient hot-casting—facilitates phase homogenization by promoting the growth of targeted *n*-phases and suppressing disproportionation [102–104]. These strategies not only improve film morphology and phase purity but also enhance the device-to-device uniformity and repeatability of RS behaviors [105]. Inclusion of such phase-engineering methodologies in quasi-2D perovskite processing could be critical for scalable integration in neuromorphic memory arrays.

While 2D and quasi-2D halide perovskites offer pronounced environmental and operational stability, primarily due to hydrophobic organic spacer cations blocking moisture and suppressing ion migration, this improvement often comes at the cost of electronic performance metrics. Specifically, the insertion of bulky spacers disrupts the continuous inorganic lattice, leading to reduced out-of-plane charge carrier mobility, increased interfacial resistance, and, in some cases, diminished device efficiency and optical absorption compared to 3D analogues [37, 89, 106]. These limitations are most evident in devices that rely on vertical charge transport, although in-plane transport may remain competitive. Nonetheless, it is important to note that all-inorganic 3D perovskites such as CsPbI_3_ can achieve remarkable environmental stability when processed under optimized conditions. Various strategies, such as partial B-site substitution, additive engineering, interface passivation, and high-temperature phase stabilization, have been shown to significantly enhance the long-term durability and operational robustness of CsPbI_3_ films and devices, sometimes rivaling those of low-dimensional materials [107–110]. Accordingly, the trade-off between stability and performance is not absolute, and continued advances in materials engineering allow for tailored selection based on application-specific requirements.

In summary, the transition from 3 to 2D halide perovskite architectures represents a fundamental advance in the quest for stable, efficient, and environmentally friendly memory materials. While 3D perovskites offer exceptional charge transport, their lack of environmental resilience and poor cycling endurance limit their practical utility. The introduction of layered structures in 2D RP and especially DJ perovskites provide a robust solution to these challenges, enabling long-term stability, suppressed ion migration, and superior device performance. The ongoing development of quasi-2D and lead-free variants further broadens the application landscape, ensuring that halide perovskites remain at the cutting edge of materials science and device engineering for years to come.

### Lead-free 2D halide perovskites

The remarkable optoelectronic properties and solution processability of lead-based halide perovskites have revolutionized the fields of photovoltaics, light-emitting diodes, and memory devices. However, the presence of lead (Pb) in these materials has raised significant environmental and health concerns, particularly as the prospect of large-scale commercial deployment becomes more realistic [111]. Lead is a well-known toxic heavy metal, and its release into the environment—whether through manufacturing, device failure, or improper disposal—poses a serious risk to human health and ecosystems. The high solubility and bioavailability of Pb^2+^ ions mean that even small amounts can accumulate in soil and water, enter the food chain, and cause neurological, developmental, and systemic disorders in both humans and wildlife. This risk is not hypothetical: recent studies have shown that perovskite solar cells and other devices can leach lead under acidic or humid conditions, and even encapsulated devices may fail catastrophically in the event of physical damage or natural disasters [112, 113]. As a result, the development of lead-free perovskite materials has become not only a scientific challenge but also an ethical imperative for the sustainable advancement of perovskite-based technologies.

In response to these concerns, researchers have explored a wide range of lead-free halide perovskites, focusing especially on two-dimensional (2D) structures that offer enhanced environmental stability and defect tolerance. The layered architecture of 2D perovskites, characterized by alternating inorganic slabs and organic spacer cations, provides a robust platform for incorporating alternative B-site metals such as tin (Sn), bismuth (Bi), and copper (Cu) [114]. These metals are generally less toxic than lead and, in many cases, are more abundant and less regulated, making them attractive candidates for sustainable device fabrication.

Among lead-free alternatives, tin-based 2D perovskites have attracted considerable attention due to the chemical similarity between Sn^2+^ and Pb^2+^ [115]. Both ions have comparable ionic radii and can form analogous perovskite lattices, preserving the desirable optoelectronic properties of their lead-based counterparts. For example, (PEA)_2_SnI_4_ exhibits a direct bandgap of 1.34 eV and strong visible-light absorption [116]. The charge carrier mobility and diffusion length in high-quality Sn-based 2D perovskite films can approach those of lead-based materials, enabling efficient charge transport in devices.

However, a critical challenge for Sn-based perovskites is the tendency of Sn^2+^ to oxidize to Sn^4+^ in the presence of oxygen and moisture [117]. This oxidation process generates deep-level defects and non-radiative recombination centers, severely limiting operational stability and efficiency. Various strategies, such as the incorporation of reducing agents (e.g., SnF_2_) and the use of inert processing atmospheres, have been employed to mitigate Sn^4+^ oxidation [118]. Recent advances in film processing, including hydrazine vapor treatment and thiourea-based passivation, have extended the air stability of Sn-based perovskites to several hundred hours, but further improvements are required for commercial viability.

Bi-based 2D perovskites represent another promising class of lead-free materials. Bi^3+^, with its stable + 3 oxidation state and relatively low toxicity, can form layered perovskite structures either as vacancy-ordered double perovskites or DJ phases [119]. The bandgap of Bi-based 2D perovskites can be tuned by doped materials. Xiao et al*.* doped CeCl_3_ to Cs_3_Bi_2_Cl_9_ [120]. The band gap of Cs_3_Bi_2_Cl_9_:Ce QDs is 3.8 eV, while that of Cs_3_Bi_2_Cl_9_ is 4.2 eV. Optimized films of Cs_3_Bi_2_Cl_9_:Ce achieve a photoluminescence quantum yield of 22.12%, representing a 71% improvement over undoped counterparts. While the charge carrier mobility in Bi-based materials is generally lower than in Sn- or Pb-based perovskites, recent work demonstrates that crystal orientation control and defect passivation can improve performance. For example, Cs_3_Bi_2_Cl_9_:Ce-based devices show fluorescence intensity enhancements by a factor of 3.2 in cellular imaging.

Copper-based 2D perovskites are a newer addition to the family of lead-free materials. Cu^2+^, when incorporated into layered perovskite structures such as (MA)_2_CuCl_*x*_Br_4−*x*_, induces strong Jahn–Teller distortions that result in broadband emission and high defect tolerance [121, 122]. These materials are notable for their environmental resilience: the organic layers act as effective barriers against moisture, and the strong metal-halide bonds resist hydrolysis and ion migration [123]. However, the indirect bandgap and relatively low charge carrier mobility of Cu-based perovskites currently limit their application in high-efficiency photovoltaics and memory devices [124, 125]. Moreover, while Cu is less toxic than lead, it can still pose ecological risks if not properly encapsulated, as Cu^2+^ ions are known to be harmful to aquatic life at elevated concentrations.

The sustainability and environmental advantages of lead-free 2D perovskites extend beyond reduced toxicity. The use of abundant and less regulated elements such as Sn and Bi alleviates concerns about resource scarcity and geopolitical supply risks associated with lead mining and refining [126, 127]. Life cycle assessments have shown that the production of Sn- and Bi-based perovskites generate significantly lower greenhouse gas emissions and hazardous waste compared to Pb-based analogs [128]. The lower processing temperatures required for 2D perovskite film fabrication (often below 150℃) further reduce the energy footprint and enable compatibility with flexible substrates and roll-to-roll manufacturing [129]. In addition, the layered structure of 2D perovskites inherently limits the leaching of metal ions into the environment, as the organic spacers act as hydrophobic barriers that slow the diffusion of water and other solvents [130]. Encapsulation strategies, such as the use of rigid spacer cations or multilayer coatings, can further enhance the environmental safety of lead-free perovskite devices, ensuring minimal metal ion release even under mechanical stress.

Despite these advantages, the transition to lead-free 2D perovskites is not without challenges. The oxidation of Sn^2+^ remains a major obstacle to the long-term stability of Sn-based devices, necessitating the development of more effective passivation and encapsulation techniques [115]. For example, SnF_2_ and SnCl_2_ additives mitigate oxidation but require precise stoichiometric control to avoid unintended decomposition [131, 132]. The scalability of high-quality 2D perovskite films, particularly those with DJ architectures, also requires further innovation in precursor chemistry, deposition methods (e.g., solvent engineering), and post-processing protocols [133]. Rapid crystallization dynamics in Sn-based perovskites often result in nonuniform films and defects, limiting device reproducibility [134]. Moreover, the environmental impact of auxiliary chemicals (e.g., hydrazine, thiourea) and solvents used in synthesis must be carefully evaluated to ensure lifecycle benefits are realized.

Recent research has focused on overcoming these challenges through a combination of materials engineering and device design. For Sn-based 2D perovskites, the introduction of Lewis base additives such as thiourea has been shown to passivate Sn vacancies and inhibit oxidation, resulting in films with improved photostability and device performance. For example, substituted thiourea ligands slow crystallization and suppress defects in 3D FASnI_3_, enhancing stability [135, 136]. Similar to 3D perovskite, the hybrid perovskite structures, such as 2D/3D heterojunctions with thiourea-based organic cations, have yielded materials with synergistic properties, including enhanced defect tolerance and environmental resilience [137]. These structures exhibit improved hydrophobicity and ion migration suppression under humid conditions.

The adoption of circular economic principles in the design and end-of-life management of lead-free perovskite devices represents another critical step toward sustainability [138]. Recycling strategies that recover Sn, Bi, or Cu from decommissioned devices can reduce demand for virgin raw materials and minimize electronic waste impacts [139]. For example, aqueous iodide solutions selectively dissolve perovskite layers, enabling > 99% metal recovery rates while preserving substrates for reuse [140]. Low-temperature pyrolysis (150–200℃) and solvent extraction methods have been demonstrated for efficient metal recovery, requiring 70% less energy than traditional lead smelting. These processes also reduce greenhouse gas emissions by avoiding high-temperature metallurgical steps. The integration of green solvents (e.g., dimethyl sulfoxide) and substrate refurbishment techniques further enhance environmental compatibility, enabling recycled devices to match the efficiency of fresh counterparts [141].

From a performance perspective, lead-free 2D perovskites have made remarkable strides in recent years. Sn-based 2D perovskite solar cells have achieved power conversion efficiencies exceeding 14.31% through interface optimization and reduced Sn^4+^ content, with ongoing improvements in stability under inert atmospheres [142, 143]. In resistive switching memory, RP phase perovskites demonstrate suppressed ion migration and enhanced moisture resistance, enabling devices with data retention over 45 days under ambient humidity [144]. While current endurance remains limited to 200 cycles for RP perovskites, DJ architectures show promise for scalability due to their compact, defect-tolerant lattices [143].

The societal and regulatory impetus for lead-free solutions is likely to accelerate the adoption of these materials in commercial products. As governments and industry stakeholders implement stricter regulations on hazardous substances in electronics (e.g., EU RoHS directives excluding photovoltaics but enforcing lead limits in other electronics), the demand for non-toxic, sustainable alternatives will only grow. Lead-free 2D perovskites, with their unique combination of environmental safety, structural stability, and electronic versatility, are well positioned to meet this demand. Their compatibility with flexible substrates, low-temperature processing, and scalable manufacturing (e.g., roll-to-roll deposition) further enhances their appeal for next-generation electronics, including wearable devices, smart textiles, and large-area memory arrays.

Despite the promise of reduced toxicity and improved environmental resilience, current Sn- and Bi-based perovskite memristors face significant challenges in terms of device stability, endurance, and overall efficiency compared to Pb-based systems. The oxidation of Sn^2^⁺ limits achievable endurance and retention times, while Bi-based perovskites suffer from intrinsically low carrier mobility and wide bandgaps. These features represent major bottlenecks for practical deployment and highlight the need for continued materials engineering to bridge the performance gap. The key performance indicators of Pb-based and various Pb-free perovskite systems are summarized in Table 2.

**Table 2:** Comparative performance metrics and limitations of Pb-based and lead-free (Sn-based, Bi-based) halide perovskite memristors

Property	Pb-based (MAPbI_3_, CsPbI_3_)	Sn-based (FASnI_3_, etc.)	Bi-based (Cs_2_AgBiBr_6_, etc.)
Endurance	~ 1,000–10,000 cycles	≤ 100 cycles (limited by Sn^2+^ oxidation)	~ 100 cycles (literature reported)
Retention	> 10,000 s	~ 1,000–10,000 s (unstable in air)	~ 1,000 s (reported)
Switching Energy	< 10 pJ	10–100 pJ	> 100 pJ (due to low conductivity)
Environmental Stability	Low (improved by encapsulation)	Unstable (oxidation, moisture-sensitive)	High (good moisture resistance)
Charge Mobility	High (~ 1 cm^2^/Vs)	Moderate to low (defects, oxidation)	Very low (< 10^−4^ cm^2^/Vs)
Ref	[61, 68, 204, 205]	[205, 206]	[148, 205]

In conclusion, the development of lead-free 2D halide perovskites represents a critical advance in the pursuit of sustainable, high-performance optoelectronic and memory devices. By replacing toxic lead with environmentally benign metals such as Sn, Bi, and copper, and by leveraging the structural advantages of layered perovskite architectures, researchers have created materials that combine the best aspects of stability, tunability, and eco-friendliness. While challenges remain in terms of efficiency, scalability, and long-term durability, the rapid progress in materials design, processing, and device integration suggests that lead-free 2D perovskites will play a central role in the future of green electronics. Continued interdisciplinary collaboration among chemists, materials scientists, engineers, and environmental experts will be essential to fully realize the potential of these materials and to ensure that the next generation of perovskite-based technologies is both high-performing and truly sustainable.

### lead-free 1D and 0D halide perovskites

In addition to 3D and 2D structures, lead-free halide perovskites have recently expanded to include one-dimensional (1D) and zero-dimensional (0D) variants. These compounds offer exceptional environmental stability and defect tolerance owing to their isolated structural motifs and limited ion migration paths, making them attractive for resistive switching applications.

1D halide perovskites, such as CsAg_2_I_3_ and K_2_CuBr_3_, consist of corner-sharing or edge-sharing metal halide octahedra arranged in linear or staircase configurations [145, 146]. These chains are spatially separated by monovalent alkali cations, suppressing long-range charge transport and ion diffusion. Such features improve resistance to ambient humidity and contribute to stable high-resistance states (HRS) in RS devices. Recent studies on K_2_CuBr_3_ memristors have demonstrated ON/OFF ratio of 10^5^ and data retention time of over 1000 s [145].

0D lead-free halide perovskites, including Cs_3_Cu_2_I_5_ and Cs_3_Bi_2_I_9_, comprise discrete metal halide clusters isolated by surrounding cations. This complete lack of dimensional connectivity results in ultra-low ion mobility and exceptional thermal and moisture stability. For example, Cs_3_Cu_2_I_5_ has been shown to exhibit strong spontaneous blue emission along with uniform bipolar RS operation with low operation voltage (within ± 1 V), ON/OFF ratio of 10^2^, stable endurance of 100 cycles, and long retention (> 10^4^ s) [147]. Similarly, Bi-based 0D devices (e.g., Cs_3_Bi_2_I_9_) have achieved retention over 10^5^ s and stable operation over 10^4^ cycles under ambient conditions [148].

Despite these advantages, there are remaining fundamental obstacles to high-speed data processing, particularly the relatively low carrier mobility and limited in-plane conductivity. Nevertheless, the demonstrated resilience and minimal ion migration in 1D/0D halide perovskites make them promising candidates for low-power, highly stable memory technologies where endurance and environmental reliability are prioritized.

## Memristors for crossbar array

### Operating principles

Resistive switching memory devices have attracted interest as promising alternatives for non-volatile memory technologies due to their distinctive features, including rapid switching speed, minimal energy consumption, scalability, and high-density data architectures. The operation of these devices relies on reversible alterations in resistance states, typically designed in a metal–insulator–metal (MIM) configuration [149, 150]. This configuration strategically places the resistive switching layer between two metallic electrodes. When exposed to controlled electrical bias, these devices are distinguished by their ability to transition between a high-resistance state (HRS) and a low-resistance state (LRS) [151–153]. The initial activation usually demands an electroforming step, during which a soft breakdown creates conductive pathways, thereby enabling the subsequent resistive modulation [154, 155]. The transition to the ON or SET state requries the application of a voltage that enables conduction via filament formation or interfacial charge accumulation. Conversely, the OFF or RESET state can be restored by applying an opposing voltage interrupting these conductive pathways [156–158].

The device performance evaluation generally involves analyzing ON/OFF current ratios, examining endurance through multiple switching cycles, and determining data retention stability over time [159–161]. Evaluation of these parameters is crucial for determining the strength and effectiveness of the devices, particularly as data demands are rapidly escalating. Bipolar resistive switching explores the various switching modes observed in resistive memory systems, especially in halide perovskite devices. This behavior is characterized by the need for polarity-specific voltages during the SET and RESET transitions, resulting in a distinctive current–voltage (*I*–*V*) hysteresis loop that explains the memory behavior [162–164]. The width of the *I*–*V* hysteresis loop is determined by the ratio of ionic to electronic mobilities; lattices where ionic motion is significantly inhibited show minimal hysteresis and enhanced accuracy in read operations. The integration of fluorinated organic spacers or Dion–Jacobson diammonium cations reduces the mobility of halide vacancies and narrows the forward-reverse current gap, thereby effectively addressing bit errors caused by hysteresis.

During a positive voltage sweep, a significant rise in current signifies the system’s shift from HRS to LRS, indicating the SET process [165, 166]. When the voltage is reversed, the current gradually drops, returning the device to HRS and completing the RESET process [167, 168]. The behavior of the cycle is essential in memory applications, as it is important for repeatedly attaining consistent and stable state changes. To reduce significant damage during abrupt current surges, particularly in electroforming and switching processes, compliance current (CC) limits are used to maintain electrical control and prevent irreversible breakdown [169–171].

Bipolar resistive switching serves as the primary operational mechanism in halide-perovskite memories, depending on the opposite direction of the electric field to switch between different resistance states [172–174]. An applied forward field promotes the creation of conductive filaments, transforming the initially high-resistance film into a low-resistance SET state. As the bias returns to a neutral state, the current decreases and an opposing polarity field subsequently disrupts the filaments, restoring the device to its high resistance RESET condition. The repetition of this pair of pulses in opposite directions allows reliable read–write cycling. Unipolar resistive switching performs SET and RESET actions using the same polarity, simplifying circuit design [175–177]. The fresh OFF state exhibits a slight leakage current until the electric field exceeds a critical threshold. The process of local heating and defect gathering establishes a stable conductive path. Maintaining the same polarity increases the temperature in the filament until it breaks, causing the film to return to the OFF state, which finishes the RESET process. The consistent voltage direction in unipolar operation minimizes crosstalk in densely packed arrays.

### Switching mechanism

The selection of materials and electrodes significantly influences switching dynamics, and a thorough comprehension of this interaction remains an active area of investigation. The fundamental principles regulating resistive switching can be broadly classified into two mechanistic categories: the electrochemical metallization (ECM) mechanism and the valence change mechanism (VCM).

**Fig. 3:**
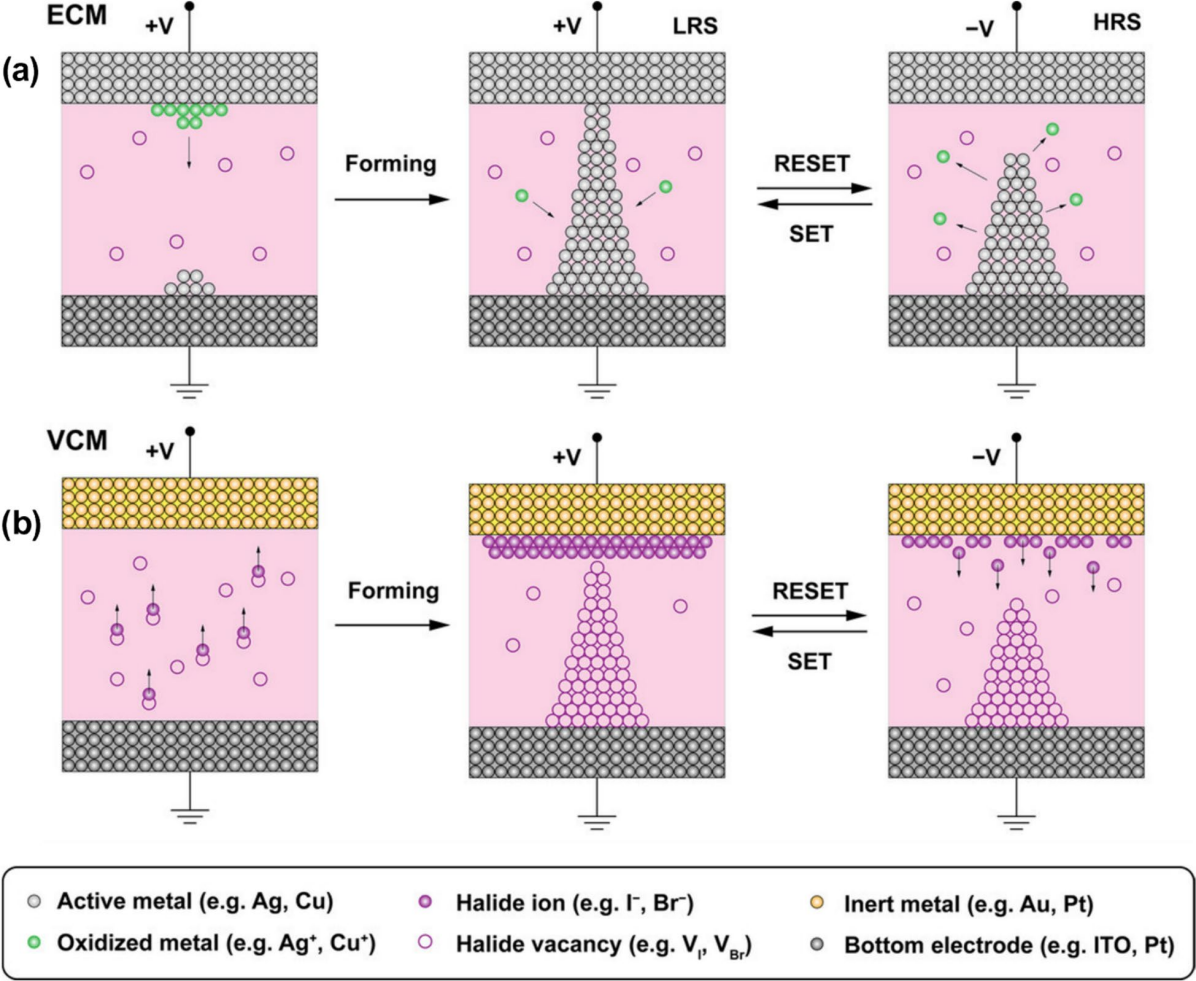
Halide perovskite-based resistive switching memory devices exhibit two principal mechanisms, **a** ECM and **b** VCM. Reproduced with permission from [186]. Copyright 2024, Wiley–VCH GmbH

In ECM-based systems, resistive switching occurs due to the movement of metal cations from an electrochemically active electrode when an electric field is applied [178, 179]. The movement of these cations across the insulating layer results in their reduction at the inert electrode, creating metallic filaments that link the electrodes and lower resistance [180–183]. These filaments dissolve when the polarity is reversed, which restores the HRS. Conversely, the VCM mechanism entails the migration of anion vacancies, including oxygen or halide vacancies, across the insulating layer [184, 185]. The existence of these vacancies affects the local oxidation states of the varying metal cations within the host lattice, thereby influencing the conductivity of the material. Resistance is modulated by valence state changes brought about by redox reactions [157]. Both mechanisms rely on the movement of ions. Nonetheless, the ECM is noted for its filamentary and generally abrupt, whereas the VCM can exhibit either filamentary or interfacial characteristics, demonstrating more gradual variations in resistance. To maximize device performance, it is critical to comprehend the primary mechanism under specific material and electrode conditions. The use of the top electrode leads to a device that demonstrates bipolar switching behavior, primarily influenced by ECM, as illustrated in Fig. 3a [186]. The SET process occurs under a low positive bias, leading to a rapid rise in current as a filament forms. Conversely, the RESET process under negative bias leads to the breakdown of the filament, returning the system to an HRS.

In contrast to devices utilizing silver (Ag), which exhibit abrupt and filamentary electrochemical metallization-dominated switching, those employing gold as the top electrode follow a different operational mechanism, as illustrated in Fig. 3b [186]. The systems demonstrate a more gradual, interface-type switching behavior, which can be related to the movement of vacancies within the switching layers. Forming these vacancies at the interface under a positive bias reduces the depletion width, enhancing charge transport, which appears as an LRS. When the polarity changes, the vacancies are distributed, resulting in an expansion of the depletion region and a rise in resistance. This VCM-based mechanism generates a more uniform switching profile that is easier to control. The gold (Au)-based device demonstrates improved modulation precision; however, it shows a higher HRS and a slightly reduced endurance than the Ag device. The difference in work function between Au and the underlying perovskite could substantially affect contact resistance and switching thresholds. Although the retention characteristics might be less durable, these systems offer important insights into the effects of interface engineering and defect dynamics on resistive switching. As a result, they form a crucial component of the broader halide perovskite-based memory research framework. Also, recent work shows that Ag–halide complex formation can couple electrochemical metallization and valence-change processes into a unified, hybrid switching pathway [187].

### Crossbar array

Memristive data storage employs two-terminal switches organized in packed crossbar arrays. An array typically consists of four fundamental components: a biased word line, a grounded bit line, a nonlinear selector that reduces sneak path currents, and the memristor itself. At every junction of the word and bit lines, the selector (S) and the resistive switching element (R) form a fully operational 1S1R memory cell [188, 189]. Programming is achieved by transferring a voltage pulse along the designated word line. When the applied potential exceeds the selector threshold, current initiates flow through the selected memristor, changing its resistance state. In contrast to memories that rely on transistors, there is no need for an extra gate electrode, enabling each cell to be accessed by merely activating the corresponding word and bit lines. The readout process involves assessing the cell resistance compared to a reference resistor, resulting in a divided voltage reflecting the stored logic level.

However, in real arrays, parasitic ‘sneak’ routes that permit current to avoid the intended cell undermine the ease of word- and bit-line addressing [190, 191]. The programming operation is relatively straightforward. As mentioned earlier, the word and bit lines are responsible for programming the cell to which they are connected. Only the target cell should focus between the selected word and bit lines so that the current can only flow through the target cell's resistance. Regrettably, *V*_*read*_ cannot be selective solely for the target cell. The design incorporates metal lines arranged in parallel, leading to multiple leakage paths that traverse the unselected cells that share these lines with the target cell [192, 193]. This results in simultaneous biasing directed at the target cell and an undefined resistor formed by neighboring unselected cells. Sneak current can disrupt the operation of a passive crossbar array, influencing the current passing through the voltage divider [193]. Recently, numerous investigations have attempted to address this issue, primarily focusing on the operational strategies to manage the unselected voltage to reduce the sneak currents.

Nonetheless, memristive devices’ inherent speed and energy efficiency advantages are compelling, driving ongoing endeavors to control sneak currents. Memristors store information in a conductance state that varies according to the total charge or flux passing through the device. Reprogramming only takes pico- to femto-joules of energy because of their small footprints, and switching takes place in less than a nanosecond. Incorporated within compact crossbar networks or organized in vertically stacked 3D arrays, each non-volatile element retains an analog value at the computation site [192–196]. At a specific junction, the current corresponds to the product of the applied voltage and the device's conductance, as Ohm’s law describes. Meanwhile, the total currents in each row come together along a column line, following Kirchhoff’s current law. This related multiply-and-accumulate functionality enables highly efficient in-memory vector–matrix processing.

A comprehensive examination of the nanoscale processes that regulate the device’s resistance is essential for understanding the origins of such remarkable performance. Memristive devices exhibit various resistance levels through unique mechanisms for each specific device type. Multiple approaches enable precise alteration of the resistance state in individual cells within a crossbar architecture. A standard method involves altering localized conductive filaments’ dimensions, volume, or composition. Alternatively, modifying the dimensions of a tunneling gap can produce analog switching characteristics; however, this often leads to nonlinear *I*–*V* behavior due to the exponential relationship between tunneling current and gap distance [192, 193]. Among these, compositional modulation within the conductive path is a promising strategy for achieving stable analog resistance states. The channel's conductance can be dynamically altered in three-terminal ionic devices via electrically controlled doping gradients induced by gate bias.

Notably, the ionic dynamics responsible for adjusting filament geometry are similar to biological learning rules, suggesting that memristors could be adequate hardware substitutes for synapses and neurons. A key feature of many memristive devices is that their conductance regulation is driven by ion migration, emulating processes seen in biological synapses and neurons. This biomimetic behavior positions memristors as attractive options for hardware-based neural networks. Their fundamental capacity to reproduce synaptic plasticity makes them suitable for various intelligent systems, encompassing image and speech processing, autonomous navigation, robotics, and biomedical diagnostics.

Therefore, precise architectural choices are necessary to translate biomimetic behavior into useful technology, particularly when comparing transistor-assisted crossbars to their denser passive counterparts. Active crossbars that employ a 1 Transistor–1 Resistor (1T1R) architecture, where each memristive element is configured in series with a MOSFET, stand out as the most promising method for realizing large-scale arrays from a manufacturing perspective [197–199]. The transistor plays a crucial role in minimizing sneak currents in unaddressed cells, which allows for accurate read and write operations. Introducing a third terminal in its gate facilitates highly linear and symmetric weight updates when applied in semi-parallel programming, thus significantly boosting on-chip training speed [193, 200, 201]. Nevertheless, the issues surrounding the area of transistors and their standby power consumption led to the exploration of an alternative method that incorporates passive crossbars, eliminating per-cell selectors to improve packing density and efficiency. This arrangement is vulnerable to half-select disturbances and sneak-path leakage, adversely affecting inference and learning processes. To address these challenges, alterations to the memristor’s current voltage characteristics are often employed, integrating notable nonlinearity or inherent rectification to reduce OFF-state conduction without additional devices[193]. Nonetheless, the resulting transfer nonidealities restrict inference to pulse-width or pulse-count modulation. An enhanced solution integrates an ultracompact selector in series with each junction, enabling the element to operate ohmically within the designated window while demonstrating precise rectification beyond that threshold. This method protects the integrity of the array while preserving real analog weight-update functionality.

To address sneak-path leakage, recent crossbar prototypes incorporate threshold-type selector devices in series with each perovskite cell, ensuring steep nonlinearity while maintaining analog weight updates. Alternative bidirectional diodes created via asymmetric band alignment within the perovskite layer offer a fundamentally selector-free approach to reducing leakage. From a fabrication standpoint, lead-free perovskite films can be deposited following front-end CMOS processing and carefully annealed, remaining comfortably within the thermal budget of back-end metal stacks. Effective surface passivation and moisture-resistant encapsulation are crucial in this process, as mobile ions must stay immobilized during the following plasma and solvent exposure in the clean-room environment. The demonstration of vertical stacking involving multiple perovskite crossbar planes has been achieved through the use of alternating ultrathin selector and memory layers. Currently, the primary scaling constraints are attributed to interlayer planarization and via resistance, rather than the active material itself. Ongoing advancements in self-aligned deposition and low-temperature etch-back are expected to facilitate the development of large-scale three-dimensional arrays, all while maintaining the integrity of selectors and the endurance of devices.

Crossbar architecture enables simultaneous connection of metal lines, resulting in unintended conduction paths among non-addressed cells that share conductors with the chosen node. The selected junction is thus suitably biased in parallel with an undetermined resistance created by the adjacent inactive cells. The current passing through that unidentified branch interferes with the voltage divider, resulting in sneak currents undermining the functionality of passive crossbar arrays lacking a selector or isolation device.

## Lead-free halide perovskite–based crossbar array

The following section details the most recent developments in lead-free halide perovskite crossbar arrays. On both flexible and wafer-scale substrates, sub-volt operation, expansive resistance windows, and excellent cycling stability have been enabled by substituting benign cations.

Su et al*.* presented lead-free cesium halide conductive-bridging memristors featuring a MoO_*x*_ interfacial layer, showing ultralow operating voltages below 0.18 V, device-to-device variations under 30 mV, retention times exceeding 10^6^ s, endurance exceeding 10^5^ complete ON/OFF cycles, ON/OFF ratios greater than 10^10^ for active areas smaller than 5 × 10^−4^ mm^2^, and over 64 distinct conductance states that maintain stability over time [86]. These characteristics facilitated stateful logic operations within a reconfigurable architecture, resulting in approximately 90% classification accuracy in a simulated handwritten digit recognition task and highlighting the potential of these devices for energy-efficient in-memory computing hardware.

The incorporation of Bi in place of lead within the halide-perovskite structure results in a material system that is both chemically stable and environmentally friendly. The resulting thin films can withstand low-temperature processing and display uniform, switchable resistive states that facilitate high-density crossbar integration. This approach creates a pathway free from heavy metals, leading to scalable, high-performance memory arrays.

**Fig. 4:**
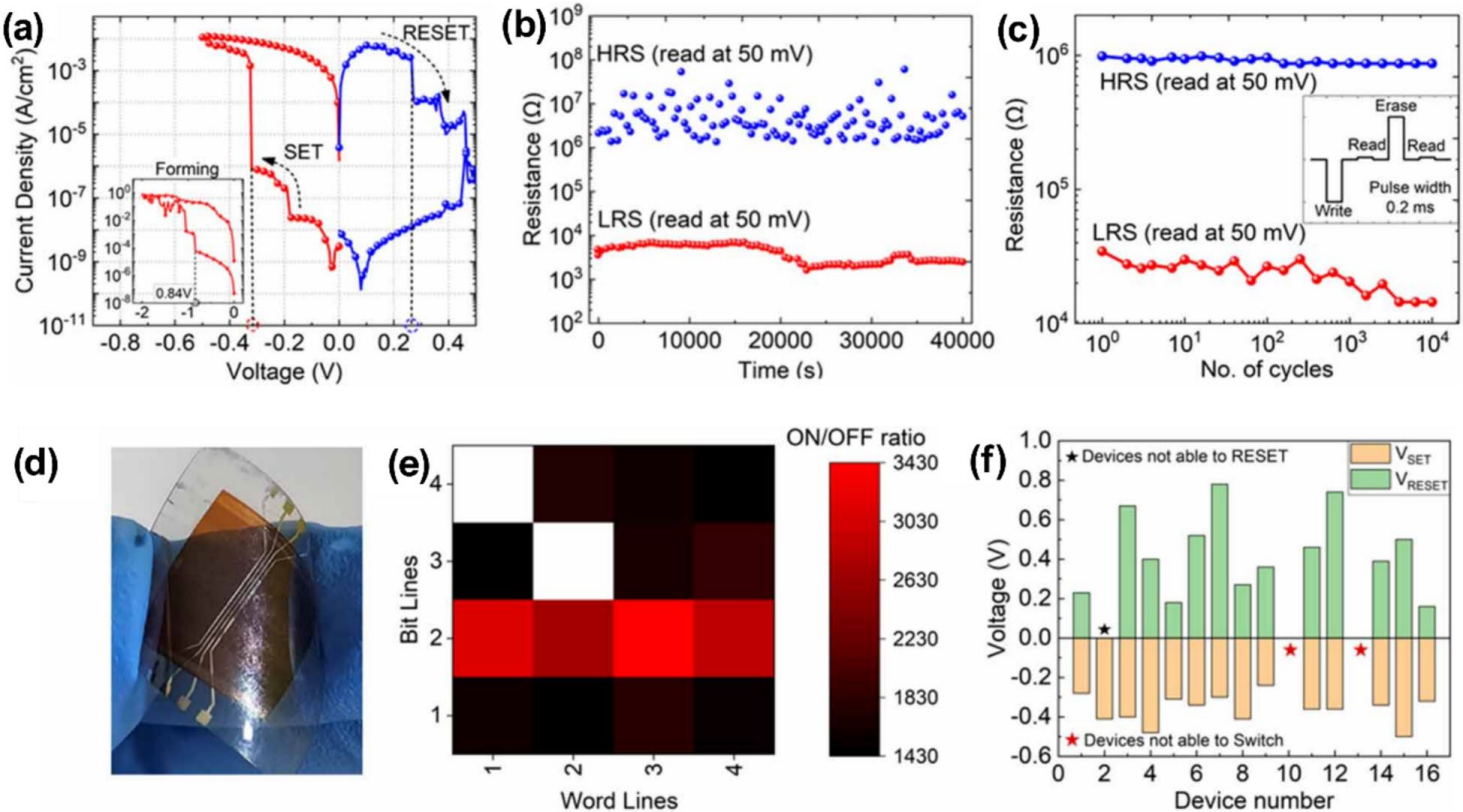
**a** The *J*–*V* curves of Cs_3_Bi_2_I_9_ perovskite-based device. **b** Retention time of Cs_3_Bi_2_I_9_ perovskite-based device. **c** Endurance cycles of Cs_3_Bi_2_I_9_ perovskite-based device. **d** Flexible 4 × 4 crossbar array fabricated on a PET substrate. **e** ON/OFF ratio map of all the devices in the crossbar array. **f** Distribution of SET and RESET voltages in the array. Reproduced with permission from [202]. Copyright 2025, IOP publishing

Yumlembam *et al.* developed flexible memristors utilizing inorganic Cs_3_Bi_2_I_9_ perovskite through hot-press and e-beam evaporation techniques [202]. The memristors made from Cs_3_Bi_2_I_9_ in this study demonstrated an initial forming requirement of around − 0.84 V, as shown in Fig. 4a. In the SET procedure, a negative sweep ranging from 0 to − 0.5 V initiated the transition from the HRS to the LRS at a SET threshold of − 0.32 V. A positive sweep ranging from 0 to 0.5 V effectively switched the element from LRS to HRS at a RESET threshold of 0.26 V. The existing ratio between LRS and HRS referred to as the ON/OFF ratio, has attained a value of 2.07 × 10^5^. Figure 4b illustrates that both resistive states exhibited stability for a duration exceeding 40,000 s (more than 11 h). The endurance evaluation utilized voltage pulses of − 0.5 V for the writing process, 0.5 V for erasing, and 0.05 V for reading. The inset of Fig. 4c illustrates a typical write-read-erase-read cycle. The device maintained an ON/OFF margin covering almost two orders of magnitude for at least 10^4^ consecutive cycles. The evaluation of large-scale integration capability involved the fabrication of a flexible 4 × 4 passive crossbar arrays, as depicted in Fig. 4d, followed by a characterization of the switching behavior of each element. The array had lamination on a 25 µm film to ensure functionality while subjected to a bending radius of 5 mm. Voltage sweeps were conducted from 0 to − 0.5 V and from 0 to 0.5 V via the word-lines, with the bit-lines held at ground potential. The bit-wise ON/OFF ratio map presented in Fig. 4e indicates that, out of the 16 cells, 13 exhibit reliable resistive switching, achieving ON/OFF ratios greater than 10^3^. The interconnected architecture demonstrates ON/OFF ratios roughly two orders of magnitude lower than those observed in stand-alone devices, a decrease that can be attributed to sneak current pathways. The statistics on operating voltage, as summarized in Fig. 4f, reveal median SET voltage and RESET voltage values of − 0.35 V and 0.40 V, respectively.

**Fig. 5:**
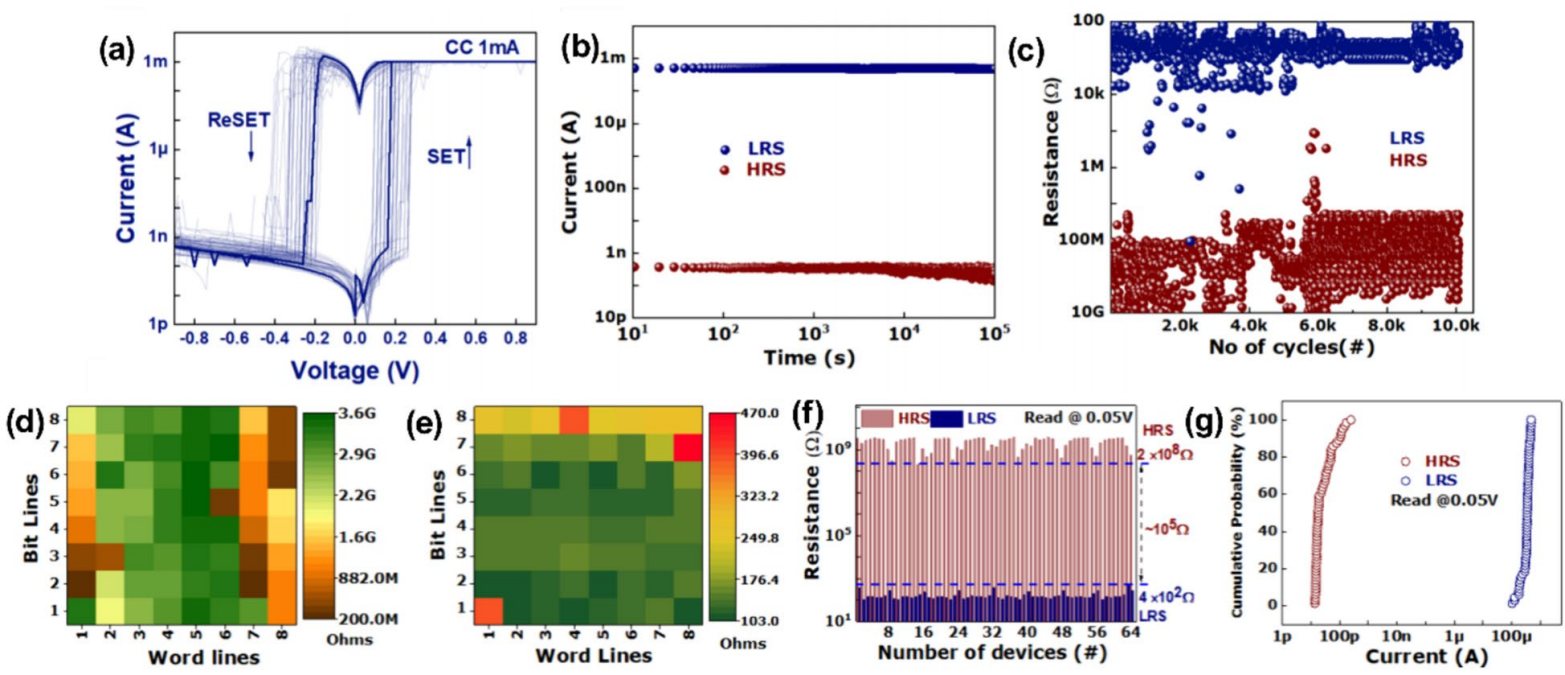
**a** 50 consecutive *I–V* characteristics of Cs_3_Bi_2_I_9_ perovskite-based device. **b** Retention time Cs_3_Bi_2_I_9_ perovskite-based device. **c** Endurance cycles of Cs_3_Bi_2_I_9_ perovskite-based device. **d** HRS distribution in color map of the 64 memristors. **e** HRS distribution in color map of the 64 memristors. **f** Resistance values of LRS and HRS. **g** Cumulative distribution of LRS and HRS values. Reproduced with permission from [148]. Copyright 2025, American Chemical Society

Also, Vishwanath et al*.* developed an 8 × 8 passive crossbar architecture utilizing Cs_3_Bi_2_I_9_ [148]. After the electro-forming process, the top electrode had a negative sweep to cause the RESET event, transitioning from LRS to HRS at − 3 V. Following this, direct-current (DC) voltage sweeps were conducted for 50 cycles, during which the array exhibited typical bipolar resistive switching behavior and a remarkable ON/OFF current ratio surpassing 10^6^. The notable ratio arises from the remarkably low leakage current in the HRS, which is due to the 0D crystal structure of Cs_3_Bi_2_I_9_ and the restricted charge diffusion pathways among the spatially isolated [Bi_2_I_9_]^3+^ octahedral complexes within the bulk lattice [148]. Highly stable bipolar switching was demonstrated by the read current (0.08 V) remaining restricted between around 0.13 nA in the HRS and 0.6 mA in the LRS over the whole set of fifty DC sweeps in Fig. 5a. Retention measurements indicated that both resistance states remained stable for over 10^5^ s under a 0.05 V read bias, showing no noticeable decay, as illustrated in Fig. 5b. Endurance examinations conducted for 10^4^ program-erase cycles consistently demonstrated reliable toggling, sustaining an ON/OFF ratio exceeding 10^4^, as illustrated in Fig. 5c. Figure 5d, e present resistance heatmaps for both the LRS and HRS within an 8 × 8 array comprising 64 cells. Figure 5f reveals a minimum HRS resistance of 2.04 × 10^8^ Ω and a maximum LRS resistance of 460 Ω. Figure 5g presents the cumulative probability distributions of current in the two states, maintaining a resistive-switching window exceeding 10^5^. The data presented exceeds the performance of earlier published halide-perovskite crossbar memristors, which experienced switching degradation within crossbar architectures. The results emphasize the strength and endurance of the 0D halide-perovskite device. Addressing sneak-path leakage in passive crossbar memories presents significant challenges without an integrated selector. Each unaddressed element was initially set to OFF in the characterization process, significantly reducing parasitic leakage. Furthermore, the spread between devices was minimal, with a significant proportion (90%) demonstrating exceptional performance, characterized by an ON/OFF ratio exceeding 10^5^.

While lead-free halide perovskites can achieve reliable resistive switching in crossbar arrays by replacing the B-site with other metal cations, adopting ordered double-perovskite phases offers an equally potent substitute. The A_2_BB'X_6_ lattice provides inherent chemical stability that supports low-voltage operation and extended data retention, expanding the possibilities for scalable, lead-free memory arrays.

**Fig. 6:**
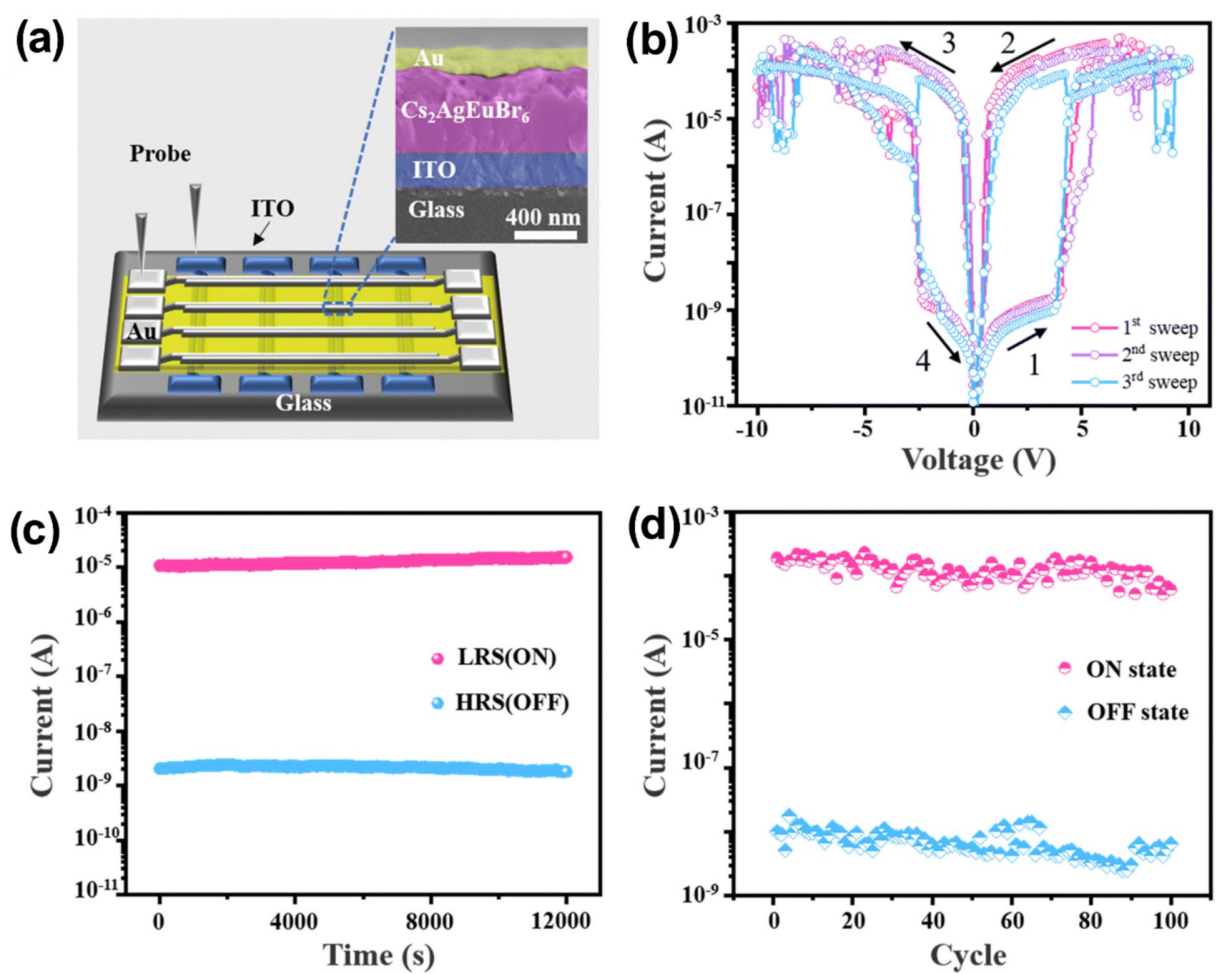
**a** Schematic diagram of a crossbar array configuration and cross-section of SEM image. **b** *I*–*V* curves with three sweeps of a memory device. **c** Retention time and **d** endurance cycles of the memory device. Reproduced with permission from [203]. Copyright 2023, The Royal society of Chemistry

Tong et al*.* reported that the lead-free double perovskite Cs_2_AgEuBr_6_, synthesized via a simple solution process, can function as the active layer. Current research on the structure of double perovskites with the Cs_2_AgBiBr_6_ lattice is considered significant, and Eu^3+^ has been suggested as an isovalent substitute for Bi^3+^ in this structural framework [203]. Figure 6a presents a schematic diagram of the fabricated Au/Cs_2_AgEuBr_6_/ITO device. Figure 6b illustrates the typical *I*–*V* characteristics. A significant current increase was observed at 3.87 V, indicating the shift from an HRS to an LRS, thus characterizing the ‘write’ process. The ON state remained consistent throughout the following sweep from − 10 V to 0 V. The transition back to the OFF state took place around − 2.38 V, marking the ‘erase’ phase, and the OFF state exhibited stability during a positive sweep from − 2.38 V to 0 V. The reliability measurement involved retention measurements and statistical analysis of HRS and LRS values across 100 devices in Fig. 6c-d. An ON/OFF ratio of 1 × 10^4^ was maintained for at least 12,000 s. Furthermore, both resistance states demonstrated narrow distributions across 100 switching cycles, highlighting remarkable uniformity.

In summary, these complementary strategies highlight the extensive design flexibility lead-free halide perovskites provide in resistive crossbar arrays. Various crystal structures and significant compositional flexibility allow for cautious adjustment of array-level switching properties to meet specific performance criteria. This adaptability establishes an effective basis for commercializing high-density, low-power, lead-free halide perovskite crossbar array memory devices.

## Conclusion

Lead-free halide-perovskite crossbar arrays are currently at the leading edge of high-density, low-power resistive memory investigations. Replacing Pb with safer cations like Sn^2+^, Bi^3+^, or Ag^+^/Eu^3+^ enables the development of Ruddlesden-Popper, Dion-Jacobson, and double-perovskite frameworks. These frameworks can withstand low-temperature solution processing, allow nanoscale patterning, and reduce toxic leakage. Flexible 4 × 4 and rigid 8 × 8 demonstrators confirm wafer-scale compatibility and emphasize potential directions for vertically stacked multilayer architectures. By addressing sneak-path leakage through compact selectors or intrinsic nonlinearity, lead-free perovskite arrays offer the potential for kilobit-class prototypes that comply with environmental regulations while maintaining speed and density. This review outlines the transition from Pb-based to lead-free halide perovskites, highlighting the role of layered chemistries in improving stability and regulatory compliance. Essential components detail the arrangements of crystals, the physics of ion migration, and the switching mechanisms that influence device performance metrics. Discussions in engineering encompass the selection of electrodes, the passivation of interfaces, the integration of selectors, and the reliability at the crossbar level. Crossbar array based on Sn-, Bi- and Ag/Eu highlights current performance levels and ongoing challenges, such as the oxidation of Sn^2+^ and variability in batch production. Final insights highlight the importance of standardized endurance tests, CMOS-compatible processing, and life cycle cost analysis as essential measures for developing manufacturable memory technologies. In the future, the field will need to go from proof-of-concept devices to fully validated, wafer-level prototypes that pass standard packaging procedures and industrial reliability tests. Important areas of investigation involve exploring the long-term behaviors of coupled ionic-electronic transport under accelerated stress, optimizing the integration of selectors with foundry back-end rules, and creating self-healing circuit architectures capable of withstanding intrinsic material variability. A comprehensive life-cycle assessment is crucial, encompassing raw material sourcing, energy consumption during processing, and end-of-life recycling, to ensure that lead-free perovskite memories evolve into a genuinely sustainable technology platform.

## Data Availability

No new data were generated in this study. All relevant information has been obtained from published sources, which are cited in the manuscript.
